# A Comparative Observational Study to Evaluate the Efficacy of Mid-Urethral Sling with Botulinum Toxin A Injection in Urinary Incontinence Patients

**DOI:** 10.3390/toxins12060365

**Published:** 2020-06-02

**Authors:** Yi-Huei Chang, Po-Jen Hsiao, Huang Chi-Ping, Hsi-Chin Wu, Po-Fan Hsieh, Eric Chieh-Lung Chou

**Affiliations:** 1 Department of Urology, China Medical University Hospital, Taichung 40447, Taiwan; yihueichang1006@gmail.com (Y.-H.C.); pojenhsiao@gmail.com (P.-J.H.); Huangchiping@yahoo.com.tw (H.C.-P.); wuhc@mail.cmuh.org.tw (H.-C.W.); 2 School of Medicine, China Medical University, Taichung 40402, Taiwan; 3 Department of Urology, China Medical University Hsinchu Hospital, Hsinchu 302056, Taiwan; 4 Department of Urology, China Medical University Beigang Hospital, Beigang, Yunlin 651012, Taiwan; 5 Graduate Institute of Biomedical Sciences, School of Medicine, China Medical University, Taichung 40402, Taiwan

**Keywords:** botulinum toxin A, mid-urethral sling, antimuscarinics, overactive bladder, urinary incontinence

## Abstract

This study aimed to evaluate and compare the efficacy and safety of mid-urethral sling (MUS) with botulinum toxin A (BoNT-A) versus MUS only in women with mixed urinary incontinence. This was a comparative observational study, and total of 73 patients were enrolled. A total of 38 and 35 patients received MUS only and MUS with BoNT-A injection, respectively. The efficacy outcome included change in Urinary Incontinence Outcome Scores (UIOS), change in Overactive Bladder Symptom Score (OABSS), and use of antimuscarinic agent or beta-3 agonist. Safety assessments included adverse events including urinary retention, increased postvoid residual volumes, and urinary tract infection. MUS with BoNT-A injection was insignificantly better than MUS only in urinary incontinence outcome (88% vs. 71%, respectively, *p* = 0.085) at week three. Among the 33 patients with detrusor overactivity (DO), patients who received BoNT-A had a higher cure rate of incontinence (88% vs. 41%, *p* = 0.01) and less required antimuscarinic agent or beta-3 agonist (31% vs. 94%, *p* < 0.001) compared to patients who did not receive BoNT-A injection. There was no significant difference in the incidences of adverse events between two groups. BoNT-A injection with MUS demonstrated efficacy and safety in the treatment of mixed urinary incontinence, specifically for women with DO.

## 1. Introduction

Urinary incontinence is a common disease observed in women, with an approximately 29–75% prevalence [1]. The common types of incontinence are stress urinary incontinence (SUI) and urge urinary incontinence (UUI). Management options for SUI include conservative treatment, pelvic floor training, and mid-urethral sling (MUS) operation. Treatments for UUI include behavioral modification and administration of antimuscarinic or beta-3 agonist agents. Furthermore, intravesical injection of botulinum toxin A (BoNT-A) is reserved for refractory patients. BoNT-A significantly decreases the number of UUI episodes and improves health-related quality of life in patients with overactive bladder (OAB) [2,3,4,5].

A high incidence of mixed urinary incontinence (MUI) is observed, or SUI and UUI coexist [6]. According to a previous study in women with MUI receiving MUS, approximately 53–79% of women experienced an improvement of UUI. However, 25–35% of women still experienced overactive bladder symptoms or de novo UUI [7]. 

We made a hypothesis that combining MUS and intravesical BoNT-A injection could have a therapeutic effect on MUI better than that of MUS alone. This study aimed to compare the efficacy and safety of MUS with or without intravesical BoNT-A injection in women who have MUI. The primary endpoints are changes in UUI episodes from baseline to week three. The secondary endpoints are add-ons in antimuscarinic agents or beta-3 agonists compared with baseline. Safety assessments included all common potential adverse events of MUS and BoNT-A intravesical injection, including urinary retention, increased postvoid residual volumes, and urinary tract infection (UTI).

## 2. Results

From July 2017 to June 2019, a total of 73 women with moderate to severe MUI were included in this observational study. The median age was 54.78 (range, 33 to 78) years. Of these, 38 patients underwent MUS only (group 1). Thirty-five patients received simultaneous MUS and intravesical injection of 80 units of BoNT-A (group 2). Detailed patient characteristics were shown in Table 1. Three months after the operation, 27 (71%) and 31 (88%) patients in group 1 and group 2 both scored 0 in the Urinary Incontinence Outcome Score (UIOS) [8] (*p* = 0.085, Table 2).

Seventeen of the 38 patients in group 1 had detrusor overactivity (DO), and 16 (94%) of them indicated that they wanted to receive treatment for OAB 3 weeks after surgery. On the contrary, 10 (48%) of the 21 patients without DO wanted to receive treatment for OAB. In group 2, 16 women had DO, and five patients wanted to receive medication treatment for OAB. In 19 patients without DO, only four patients wanted to receive medication treatment (Table 3).

The Overactive Bladder Symptom Scores (OABSS) in the MUS only group before and after 3 weeks of management were 8.5 and 6.1, which were not statistically different (*p* = 0.084). The OABSS in the MUS with BoNT-A group were 9.3 before surgery and 3.5 after 3 weeks of surgery, which showed significant improvement (*p* < 0.001, Table 4). Three months after surgery, patients with persistent bladder symptoms have been treated with oral medications, and the OABSS in groups 1 and 2 were 4.2 and 3.3, respectively.

Based on further analysis of the data on week 12 after surgery, it was found that DO and OABSS ≥11 (area under the curve = 0.96, *p* = 0.003, 95% confidence interval, 0.893–1.000) were predictors of successful treatment when MUS was combined with BoNT-A injection (Table 5).

The complication rates between the two groups were similar (Table 6). In the MUS with BoNT-A group, eight of the 35 patients complained of difficulty in urination. Six of them showed impaired detrusor contractility (bladder contractility index <100) in the urodynamic study before surgery [9]. These symptoms improved 12 weeks after the operation.

## 3. Discussion

Urinary incontinence has a considerable impact on quality of life and significantly affects morbidity [10,11]. In women with urinary incontinence, approximately 50% and 30–40% have SUI and MUI, respectively [12]. Coexistence of SUI and UUI could increase the severity of leakage and significantly affect the patients’ quality of life. Besides nonsurgical management, MUS for SUI is considered significantly effective in the treatment of storage symptoms [13,14]. On the contrary, conservative treatments for UUI mainly include behavioral modification, pelvic floor muscle training, and administration of oral medication [15]. New treatment modality should be considered considering that there is no single treatment applicable to treat both symptoms simultaneously.

Clinically, treatment decision will be determined according to the predominance of SUI or UUI. For example, if SUI is predominant, MUS surgery will be arranged, and patient may receive antimuscarinic agent after surgery [13,14]. However, some studies have pointed out that surgery for SUI is considered not beneficial or can even worsen the symptoms of OAB [16,17]. Moreover, approximately 6–8% of women treated with MUS will develop de novo OAB. On the contrary, if patient’s symptom is UUI predominant, surgery is generally not recommended. Antimuscarinic agent and beta-3 agonist are administered to treat symptoms, but these patients often still experience persistent urinary leakage because of SUI. It is difficult for women to evaluate themselves whether SUI or UUI is more predominant. In some of these women, only SUI is treated; thus, they still experience UUI.

Intravesical injection of BoNT-A is also relatively effective for OAB-wet and has been approved by the Food and Drug Administration [18]. To the best of our knowledge, this is the first study to compare MUS with concomitant BoNT-A intravesical injection with MUS only to treat MUI. If two types of urinary incontinence can be treated simultaneously, it should be a good choice for patients. This study successfully expanded the clinical indication of BoNT-A in treating MUI.

Our results show that the continence rate of patients with moderate to severe MUI who received BoNT-A while undergoing MUS was slightly better than that of patients receiving MUS only (cure rate 88% vs. 71%, respectively), but the difference was statistically insignificant. More importantly, not only the symptoms of urinary leakage but also the symptoms of OAB improved in patients receiving both MUS and BoNT-A intravesical injection.

The possible complication of urinary retention is a great concern of patients with MUI receiving MUS and BoNT-A intravesical injection simultaneously. Sun et al. reported that the complication risks of 100 units of BoNT-A intravesical injection included UTI (35%) and urinary retention (8–10%) [18]. In our previous pilot study, five patients received 100 units of BoNT-A intravesical injection. All of them experienced difficulty in voiding and had postvoid residual urine greater than 150 mL. Moreover, three of these five patients required single catheterization after receiving MUS combined with 100 units of BoNT-A intravesical injection. Kuo et al. reported that the range of BoNT-A dosage from 50 U to 300 U showed significant improvement in OAB and urinary incontinence and in urodynamic measures, but receiving >100 units of BoNT-A (*p* = 0.029) was considered a predictor for the increasing incidence of adverse event such as straining to void [19]. Thus, we decreased the BoNT-A dosage to 80 units in this study, and patients did not experience urinary retention. Although 23% of the patients had transient difficulty in urination, these symptoms all improved 12 weeks after the operation.

We found that using a lower dose of 80 units of BoNT-A prevents urinary retention. Previous studies have pointed out that the lower BoNT-A dose has shorter efficacy [20]. Hence, proper explanation regarding the possible reinjection of BoNT-A in 3 months after surgery was provided to patients before surgery. We comprehensively discussed with the patients whether they needed to receive another BoNT-A intravesical injection or to undergo behavioral therapy combined with the administration of oral medication. Finally, 35% of patients decided to continuously receive BoNT-A intravesical injection.

There are several ways to treat urgency symptoms in MUI such as behavioral therapy, pelvic floor muscle training, and administration of oral medication [21]. Most MUI patients initially receive oral medications for their symptoms. However, these oral medications such as anticholinergic agents or beta-3 agonists have significant side effects. Side effects of anticholinergic agents include dry mouth, constipation, and cognitive problems in the elderly [22]. Although beta-3 agonists have relatively lesser side effects than anticholinergic agents, blood pressure may increase in some patients after receiving beta-3 agonists, and the long-term effects of beta-3 agonists on the elderly are still unclear [23,24]. In our study, only 26% continued to take the medication for OAB. Therefore, MUS combined with BoNT-A intravesical injection is considered beneficial in reducing the side effects of oral medications.

In our study, decreasing urination flow rate after surgery was associated with MUS or BoNT-A intravesical injection. However, none of the patients required urinary catheterization in our study. Moreover, adjusting the tension of the sling was not needed while we performed MUS surgery combined with BoNT-A intravesical injection, although BoNT-A may strongly reduce the contractility of the bladder. Previous studies pointed out that the mechanism of MUS was supported by a “tension-free” method instead of urethral obstruction [25].

In our study, all of the patients experienced MUI, but the proportion of DO that was observed during the urodynamic examination before surgery was 45%. According to the definition of the ICS [26], the diagnosis of UUI is based on the patient’s symptoms. During the preoperative evaluation, the patient stated that it was difficult to distinguish whether SUI or UUI was predominant. For example, during certain movements, such as brisk walking or small jogging, increased abdominal pressure was accompanied by urgency to leak urine. In our study, we found that in patients who received both treatments, the symptoms still improve in patients without DO. However, patients with DO receiving MUS alone had higher risk of persistent UUI after surgery than patients receiving MUS combined with BoNT-A intravesical injection. We recommend that patients with MUI combined with DO should appropriately receive MUS and BoNT-A intravesical injection.

There were some limitations to this study. First, this was an observation study instead of a randomized controlled trial. According to the guideline of European Association of Urology and American Urological Association, both MUS and BoNT-A intravesical injection are standard treatments for urinary incontinence. In preoperative counseling we explained to every patient that BoNT-A intravesical injection carried 5% incidence of urinary retention and clean intermittent catheterization might be necessary [5]. Based on patients’ autonomy, we let them choose whether to receive the BoNT-A intravesical injection or not. There was no significant difference in age, BMI, and OABSS between the two groups. Second, the case number was small. In addition, currently there is no objective and universally accepted tool to evaluate treatment outcomes of MUI. Nevertheless, the promising results shown in OABSS and UIOS improvement as well as freedom from OAB medications could support the rationale to conduct a prospective, large-scaled, randomized study to confirm the efficacy and safety of MUS combined with BoNT-A intravesical injection in treating MUI. 

## 4. Conclusions

An 80-unit BoNT-A injection combined with MUS is not only effective but also safe in the treatment of MUI patients. DO and high OABSS are predictive factors for a satisfactory treatment outcome in patients.

## 5. Materials and Methods

This retrospective observational comparative study was done in a tertiary referral center. The inclusion criteria were women who had at least one episode of SUI and one episode of UUI in a 3-day voiding diary. Patients with urethral diverticulum, urinary fistula, previous urinary incontinence surgery, intravesical BoNT-A injection, or pelvic floor reconstruction, and history of neurogenic bladder were excluded. If they had previously taken medications for OAB, a 3-week washout period was required before surgery. This study was approved by the Ethic Committee of China Medical University Hospital, and the protocol number was DMR-94IRB-083(FR) (Approval date: 30 November 2016).

Preoperative evaluation including history assessment, physical examination, urinalysis, urodynamic study, and assessment of OABSS and UIOS were done at the outpatient clinic. The decision of MUS only or MUS with BoNT-A intravesical injection was made by the patient after a detailed explanation of the procedure and morbidities. All patients were reassessed at 1 week, 3 weeks, 3 months, and 6 months after surgery. According to the UIOS, cure, improvement, and failure scored 0, 1–4, and 5, respectively. In our study, a score ranging from 1–5 was defined as the absence of cure [8].

The patients in the MUS only group underwent a surgical procedure performed by a single surgeon (ECL Chou) using a transobturator MUS (Contasure-KIM^®^, Neomedic International, Leganés, Madrid, Spain). The patients in the MUS with BoNT-A group received transobturator MUS and 80-unit BoNT-A(BOTOX^®^, Allergan, Irvine, CA, USA) intravesical injection.

During a 3-week evaluation after surgery, patients in both groups were asked if they wanted to receive medication treatment for OAB to control the symptoms of urgency, frequent urination, and urgency incontinence. Six months after the operation, patients who had received BoNT-A intravesical injections were also evaluated if they wanted to repeatedly receive BoNT-A intravesical injection.

All comparisons of patients’ categorical characteristics and outcomes were assessed using the Fisher’s exact test. Mann-Whitney U test was used to compare the means of continuous variables such as OABSS in two groups. We compare continuous data before and after intervention by Wilcoxon signed rank test. The predictive factor of successful treatment was evaluated by mixed linear regression. All statistical assessments were performed by two-sided analysis, and significant differences were considered at a *p*-value < 0.05. The Statistical Package for the Social Sciences (SPSS) version 19.0 statistical software (SPSS Inc., Chicago, IL, USA) was used in all statistical analyses.

## Figures and Tables

**Table 1:** Patient characteristics.

	MUS Only (n = 38)	MUS with BoNT-A (n = 35)	*p* Value
Age, median (years)	53.70 ± 11.10	55.30 ± 12.40	0.690
Body mass index, median (kg/m^2^)	25.30 ± 2.40	26.20 ± 2.70	0.230
Parity, mean	2.60 ± 1.00	2.80 ± 0.8	0.390
OABSS, mean	8.47 ± 2.44	9.31 ± 2.37	0.084

MUS: Mid-urethral sling, BoNT-A: botulinum toxin A, OABSS: Overactive Bladder Symptom Score.

**Table 2:** Surgical outcomes of the study population.

	MUS Only (n = 38)	MUS with BoNT-A (n = 35)	*p* Value
Cure (UIOS = 0)	27 (71%)	31 (88%)	0.085
Need medication for OAB after operation	26 (68%)	9 (26%)	<0.001

UIOS: Urinary Incontinence Outcome Score, OAB: overactive bladder.

**Table 3:** Surgical outcomes of patients with and without detrusor overactivity.

	MUS Only	MUS with BoNT-A	*p* Value
Patients with DO	17	16	1.000
Cure (UIOS = 0) after operation	7 (41%)	14 (88%)	0.010
Need medication after operation	16 (94%)	5 (31%)	<0.001
Patients without DO	21	19	1.000
Cure (UIOS = 0) after operation	20 (95%)	17 (89%)	0.596
Need medication after operation	10 (48%)	4 (21%)	0.105

DO: detrusor overactivity, UIOS: Urinary Incontinence Outcome Score.

**Table 4:** Evaluation of overactive bladder symptoms score at three weeks.

OABSS	Pre-Operation	Post-Operation
MUS only (n = 38)	8.5	6.1
MUS with BoNT-A (n = 35)	9.3	3.5
*p* value	0.084	<0.001

OABSS: Overactive Bladder Symptoms Score.

**Table 5:** Multiple linear regression analysis between successful treatment and factors.

Factors	Beta Coefficient	95% CI		
		Lower Bound	Upper Bound	*p* Value
OABSS	0.099	0.052	0.147	<0.001
DO	−0.505	−0.532	−0.113	0.004

DO: detrusor overactivity, OABSS: Overactive Bladder Symptoms Score.

**Table 6:** Adverse events 3 weeks after operation.

Variable	MUS Only	MUS with BoNT-A	*p* Value
Urinary tract infection	5 (13%)	9 (26%)	0.237
Bladder perforation	0	0	N/A
Tape exposure	0	0	N/A
Acute urinary retention	0	0	N/A
Large PVR (>150 mL)	4 (11%)	7 (20%)	0.334
Difficulty in urination	6 (16%)	8 (23%)	0.556

PVR: post voiding residual urine volume.

## Abbreviations

The following abbreviations are used in this manuscript:

MUS	mid-urethral sling
BoNT-A	botulinum toxin A
UIOS	Urinary Incontinence Outcome Scores
OABSS	Overactive Bladder Symptom Score
UUI	urge urinary incontinence
SUI	stress urinary incontinence
MUI	mixed urinary incontinence
OAB	overactive bladder
DO	detrusor overactivity
